# Immune Regulator Retinoic Acid-Inducible Gene I (RIG-I) in the Pathogenesis of Cardiovascular Disease

**DOI:** 10.3389/fimmu.2022.893204

**Published:** 2022-05-26

**Authors:** Hao Wang, Jie Yin, Xinyan Gu, Wenhui Shao, Zhanjun Jia, Hongbing Chen, Weiwei Xia

**Affiliations:** ^1^ Department of Clinical Laboratory, Children’s Hospital of Nanjing Medical University, Nanjing, China; ^2^ Department of Cardiology, Children’s Hospital of Nanjing Medical University, Nanjing, China; ^3^ School of Pediatrics, Nanjing Medical University, Nanjing, China; ^4^ Department of Nephrology, Children’s Hospital of Nanjing Medical University, Nanjing, China; ^5^ Jiangsu Key Laboratory of Pediatrics, Nanjing Medical University, Nanjing, China

**Keywords:** RIG-I, activators, signal pathway, inflammation, cardiovascular diseases

## Abstract

Retinoic acid-inducible gene I (RIG-I) is a cytosolic pattern recognition receptor that contains two CARD domains, an RNA helicase domain, and a C-terminal domain. RIG-I initiates antiviral innate immunity by recognizing exogenous viral RNAs/DNAs. However, some studies have reported that RIG-I activation leads to damage in various organs and tissues in diverse circumstances. Recent studies have shown that RIG-I is involved in cancer, lupus nephritis, immunoglobulin A nephropathy, Crohn’s disease, and atherosclerosis. These reports indicate that RIG-I not only participates in antiviral signaling pathways but also exerts an influence on non-viral infectious diseases. RIG-I is widely expressed in immune and non-immune cells including smooth muscle cells, endothelial cells, and cardiomyocytes. A succinct overview of RIG-I and its signaling pathways, with respect to the cardiovascular system, will aid in the development of novel therapeutics for cardiovascular diseases. In this review, we summarize the structure, activation, signaling pathways, and role of RIG-I in cardiovascular diseases.

## Introduction

The innate immune response serves as the first line of defense against pathogens and transfers signals to activate the adaptive immune system to eliminate invading pathogens (1). Short-term activation of the innate immune system is beneficial for the elimination of pathogenic microorganisms, and tissue repair. However, sustained or excessive innate immune activation is unfavorable and detrimental to organs (2–4). Pattern recognition receptors (PRRs) expressed in the innate immune cells mediate innate immune responses (5) and accelerate inflammation. PRRs are divided into four groups: Toll-like receptors (TLRs), RIG-I-like receptors (RLRs), NOD-like receptors (NLRs), and C-type lectin receptors (CLRs) (6). RLRs play a crucial role in recognizing viruses and triggering inflammation (7).

RLRs include MDA5 (melanoma differentiation-associated factor 5), LGP2 (laboratory of genetics and physiology 2), and RIG-I (retinoic acid-inducible gene I) (8). RIG-I is the first identified RLR and is induced by all-trans retinoic acid in acute promyelocytic leukemia cells (9). Thus far, RIG-I has been of interest and explored; it can detect RNA virus infection and induce production of interferon (IFN), inflammatory cytokines, and chemokines (10) *via* stimulation of transcriptional factors, including interferon regulatory factor (IRF), nuclear factor-κB (NF-κB), and activator protein-1 (AP-1). To date, the structure, activation, signaling pathways, and function of RIG-I in innate immunity have been well documented (11). However, many new insights into the other biological functions of RIG-I have emerged to extend the role of RIG-I as a PRR. Accumulating evidence has shown that RIG-I participates in cellular damage and the occurrence and development of many diseases, such as acute myeloid leukemia (AML), hepatocellular carcinoma, lupus nephritis, immunoglobulin A nephropathy, Crohn’s disease, rheumatoid arthritis, and cardiovascular diseases (CVD) (12–18).

CVD is one of the main causes of death worldwide and imposes a heavy economic burden on families and society (19). The pathogenesis of CVD is complex. It involves many pathological processes including endothelial cell dysfunction, proliferation and migration of vascular smooth muscle cells (VSMCs), apoptosis, cardiomyocyte hypertrophy, fibrosis, and heightened inflammatory response. Experimental studies have focused on the effect of RIG-I-mediated inflammation in the development and complications of human cardiovascular diseases (20), indicating the potential of RIG-I as a therapeutic target in the treatment of cardiovascular diseases. This review elucidates the role of RIG-I in the etiology of cardiovascular dysfunction and the pathogenesis of cardiovascular disease.

## The Structure of RIG-I

Human RIG-I is encoded by DDX58, which maps to chromosome9p21.1 and comprises 18 exons. RIG-I is a cytosolic protein containing 925 amino acids and the length of its mRNA is 2775 bp (21). It is a member of the RLR family and contains two N-terminal caspase active recruitment domains (CARDs), a catalytic helicase core consisting of two RecA-like domains (Hel1 and Hel2), and a C-terminal domain (CTD) (22, 23). The two N-terminal CARDs are essential for initiating downstream antiviral signaling molecular transduction by binding to the mitochondrial antiviral signaling protein (MAVS). The catalytic helicase core has ATPase and translocase activities, which are essential for binding RNA and catalyzing ATP hydrolysis. The C-terminal domain (CTD), also known as the repressor regulatory domain (RD), is necessary for RNA-terminus recognition (24–26).

## The Activation of RIG-I

As a key intracellular viral RNA sensor, RIG-I is activated by short (<300bp) double-strand RNA and 5’-triphosphate single-strand RNA to promote the formation of interferons that trigger and mediate antiviral responses (27–29). RIG-I can specifically distinguish cytosolic viral dsRNAs from self-RNAs, with its ATPase activity playing a crucial role in this discrimination (30). Self RNAs do not activate RIG-I signaling because their 5’ppp is capped by 2’O-methylation (31, 32). Moreover, deficiencies in these aspects cause autoimmune disease *via* self-RNAs that activate RIG-I signaling (33, 34). Other studies have shown that RIG-I can detect single-strand RNA (ssRNA) viruses to mediate antiviral responses during infection (28, 35, 36). Saito et al. found that the hepatitis C virus could also be detected by RIG-I *via* binding to the A/U-rich motif in the 3′-untranslated region of the genome (37). In addition to viral RNAs, many analogs of double-stranded RNA, including poly(I: C) and poly(A: U) are specifically recognized by RIG-I (38). A recent study reported that mitochondrial RNA triggers a RIG-I-MAVS-dependent immune response (39).

Several DNA sensors including TLR9, AIM2, and cGAS have been identified (40–42). RIG-I, a cytosolic RNA receptor, also recognizes cytosolic DNA to selectively activate the expression of type I IFN genes (43). Furthermore, studies have shown that apart from RNA/DNA, lipopolysaccharide (LPS) (44), interferon-gamma (45), interleukin (IL)-1β (46), and TNF-α (47) also activate RIG-I signaling to mediate the inflammatory response. Furthermore, RIG-I mediates LPS- or IFN-γ-induced inflammation in endothelial cells and vascular smooth muscle cells, indicating that RIG-I is crucial in non-antiviral inflammation-related diseases. Considering the pathogenic roles of these new RIG-I activators in the elderly, patients in ICU, patients with organ transplantation, and patients with immune deficiency (48–51), targeting RIG-I could be a therapeutic option for these patients.These activators of RIG-I are summarized in **Table 1**. The above-mentioned studies indicate the pleiotropic functions of RIG-I.

**Table 1:** Activators of RIG-I.

Categories	Activators
RNA	dsRNAs; 5'-triphosphate single-stranded RNA; poly I:C; poly(rU):poly(rU) RNA; mitochondrial RNA
DNA	DNA
Others	LPS; IFN-γ; IL-1β; TNF-α

### The Antiviral Signaling Pathway of RIG-I

Studies on virus infection provide fundamental information on the RIG-I signaling pathway. RIG-I is an auto-regulated protein that exhibits auto-inhibition of the interaction between the CTD and CARD domains. During viral infection, viral dsRNA binds to the CTD domain and the N-terminal CARD is exposed for downstream signaling (23). RIG-I then interacts with the adaptor protein MAVS through a CARD-CARD interaction. MAVS is also known as a virus-induced signaling adaptor (VISA), CARD adaptor inducing IFN-β (Cardif), and IFN-β promoter stimulator (IPS-1). MAVS is located in the mitochondrial outer membrane *via* its C-terminal transmembrane (TM) domain (52). It interacts with RIG-I *via* CARD-CARD domains (53).

The RIG-I/MAVS signaling pathway is divided into two branches, with one branch inducing the production of type I interferons and other inducing the production of pro-inflammatory cytokines. During the production of type I interferons, MAVS recruits TANK-binding kinase 1(TBK1) and inhibitor of κB kinase (IKKϵ) to phosphorylate the transcription factors, interferon regulatory factors IRF-3 and IRF-7, to phosph-IRF3 and phosph-IRF7, respectively. The phosphorylated factors translocate to the nucleus and induce the production of type I interferons (54). On the other branch, MAVS recruits IKKα, IKKβ, and IKKγ to induce the phosphorylation and destruction of IκBs for the activation of nuclear factor ‘kappa-light-chain-enhancer’(NF-κB), which promotes the expression of pro-inflammatory cytokines (55).

**Figure 1:**
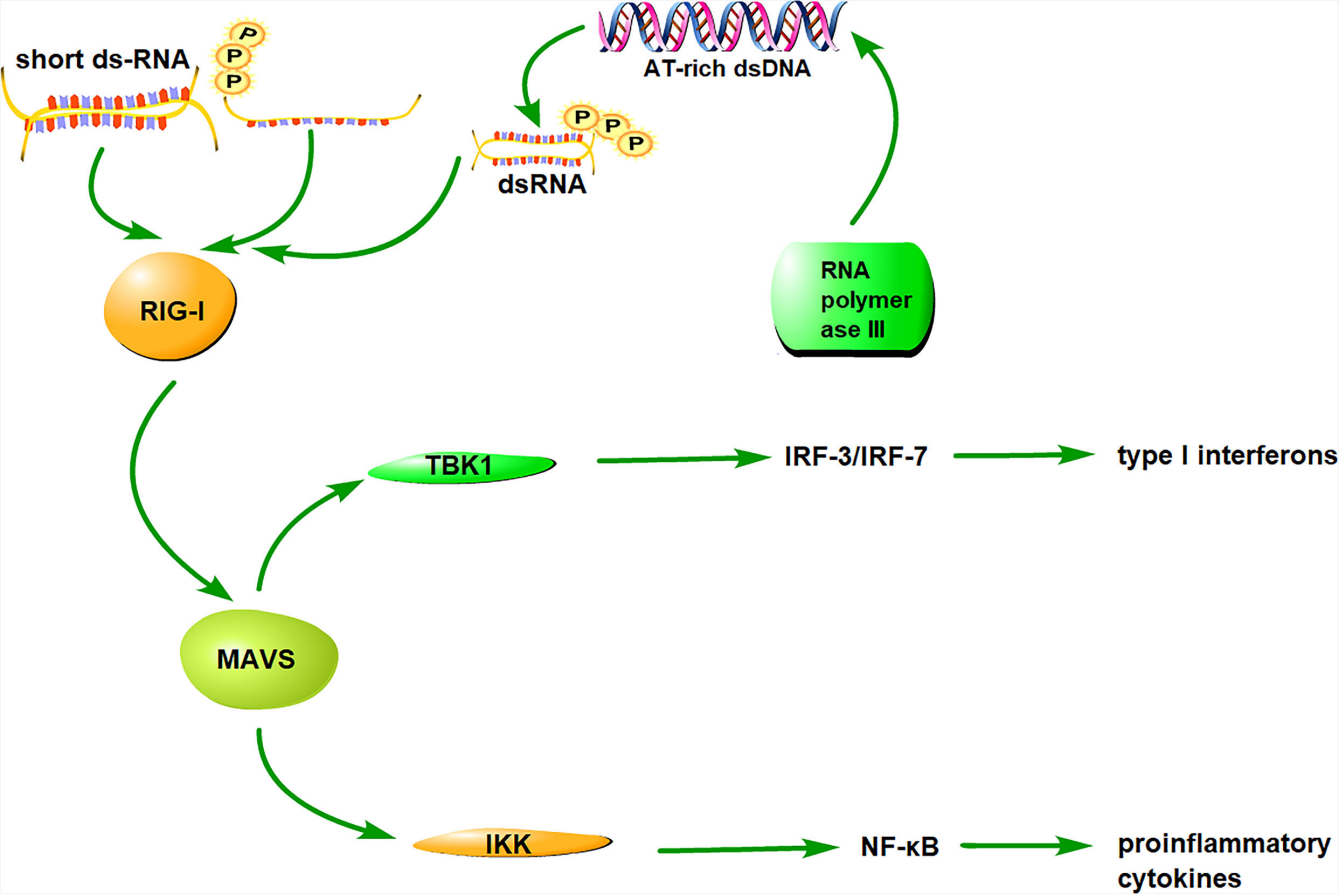
The signaling pathway of RIG-I. RIG-I distinguishes and binds to RNA/DNA *via* the CTD, subsequently exposing CARDs and catalyzing ATP hydrolysis. RIG-I interacts with its downstream adaptor molecule, MAVS, and activates two cytosolic protein kinase complexes, TBK1 and IKK. The TBK1 complex phosphorylates IRF-3/7 and induces type I interferon production, whereas the IKK complex activates NF-κB and promotes the production of proinflammatory cytokines.

Unlike the RNA-RIG-I pathway, the DNA-RIG-I pathway can interact directly with DNAs or an RNA intermediate derived from DNA through RNA polymerase III transcription (43, 56). The recognition of DNAs or DNA-derived RNA intermediate depends on the type of cell line and the structure of the DNA and DNA-derived RNA. The RNA-RIG-I pathway can activate IRF3/7 and NF-κB for inducing production of type I interferons and pro-inflammatory cytokines, respectively; whereas, the DNA-RIG-I pathway primarily activates the transcription factor IRF3 to generate type I IFNs. These results indicate the diverse role of RIG-I in the response to RNA- or DNA-containing pathogens. The RIG-I antiviral signaling pathway is shown in **Figure 1**.

### RIG-I in Regulating the Function of Cardiovascular Cells and Macrophages

It is known that both the injury of the cardiovascular cells and the activation of inflammatory cells contribute to the pathophysiology of cardiovascular system. During the occurrence and development of cardiovascular diseases, RIG-I in cardiovascular cells and macrophages was reported to be of importance in the disease pathology.

### RIG-I in Endothelial Cell Dysfunction

The endothelium, which lines the interior surface of blood vessels plays an important role in controlling vascular permeability. Vascular endothelial cells have an essential function in restraining inflammation and avoiding thrombosis (57). Thus, they play a critical role in acute and chronic inflammation (58, 59). Vascular endothelial cells participate in immune and inflammatory reactions by inducing the expression of various cytokines and adhesion molecules. Additionally, the inflammatory response of endothelial cells leads to a pro-thrombotic state (coagulopathy, increased vascular permeability, arterial hypotension, and organ dysfunction) and increases the risk of cardiovascular diseases (60).

Studies have shown that RIG-I activates innate immunity and inflammation to promote endothelial cell (EC) dysfunction. Dengue virus (DENV) induces RIG-I activation in microvascular endothelial cells to increase the production of type I IFN, ICAM-1, and other pro-inflammatory cytokines, resulting in endothelial injury (61). In porcine circovirus disease (PCVD), porcine circovirus type 2 (PCV2) upregulates the production of inflammatory factors in arterial endothelial cells *via* the RIG-I signaling pathway, which eventually leads to endothelial dysfunction and vascular system disorders (62). In addition, RIG-I activation by RIG-ligand 3p-RNA induces endothelial damage by enhancing reactive oxygen species (ROS) formation and pro-inflammatory cytokine release, contributing to atherogenesis (63). Poly (I:C), an analog of double-stranded RNA, impairs sodium nitroprusside (SNP)-induced rat superior mesenteric artery relaxation by activating the RIG-I/NF-κB/iNOS pathway (64). The above studies suggest that the antiviral process mediated by RIG-I signaling could be accompanied by inflammatory injury in endothelial cells. Thus, it is imperative to consider targeting RIG-I as a therapeutic strategy for the treatment of virus-related diseases.

As mentioned previously, RIG-I activation by viral RNA or RNA analogs induces endothelial damage. RIG-I activation by non-viral ligands also plays an important role in mediating endothelial injury. For example, LPS induces the expression of RIG-I in endothelial cells, and RIG-I overexpression selectively upregulates the expression of COX-2, which participates in inflammation and vascular injury (44). Moreover, LPS activates RIG-I to upregulate the expression of proinflammatory molecules in endothelial cells to mediate sepsis (65, 66). In addition, Imaizumi et al. observed that IFN-γ induced RIG-I expression, which mediated immunological reactions and inflammatory responses in HUVECs, leading to endothelial damage (45). Furthermore, Wang et al. showed that 25-hydroxycholesterol promoted inflammation in HUVECs *via* the IRF1/RIG-I axis, which contributed to atherosclerosis (20). These results indicated that activation of RIG-I by non-viral ligands promotes endothelial damage by enhancing the inflammatory response. Moreover, in addition to the effect of RIG-I on inflammation-mediated endothelial dysfunction, a study demonstrated the effect of RIG-I on the pro-thrombotic state. They found that dsDNA poly(dA:dT) and hepatitis B virus induced the expression of prothrombotic proteins in vascular endothelial cells, which accelerated microvascular thrombus formation *in vivo* and promoted upregulation of von Willebrand factor (vWF) and platelet tethering *via* RIG-I signaling (67).

Taken together, these studies demonstrate that RIG-I activation leads to endothelial cell injury and dysfunction by enhancing inflammation and thrombosis. Blockade of RIG-I signaling in non-viral diseases associated with inflammatory injury of endothelial cells might be beneficial for maintaining the integrity of the endothelium.

### RIG-I in Vascular Smooth Muscle Cell Dysfunction

Smooth muscle cells (SMCs) are one of the major components of the vascular wall and involved in vascular disorders such as vasospasm, hypertension, and atherosclerosis. Proliferation, migration, dedifferentiation, and apoptosis of vascular SMCs contribute to the pathogenesis of vascular diseases (68). A recent report showed that IFN-γ induced RIG-I expression in SMCs *in vivo* and *in vitro* (69). A previous study also found that G3BP1 interacts with RIG-I and further activates MAVS to act on aortic SMCs and drive aortic calcification. Accordingly, a G3BP antagonist downregulated RIG-I-stimulated G3BP1 methylation; hence, RIG-I and MAVS deficiency reduced osteogenic signals in VSMCs, attenuating arteriosclerosis (70). Another study showed that lncRNA growth-arrest-specific transcript 5 (GAS5) induced SMC apoptosis and subsequent abdominal aortic aneurysm (AAA) by activating the zeste homolog 2 (EZH2)-mediated RIG-I signaling pathway in angiotensin II-induced AAA mouse models (71). These evidences highlight that RIG-I activation contributes to SMC dysfunction and vascular diseases, including aortic calcification and abdominal aortic aneurysms. Further research on the detailed mechanisms of RIG-I in SMC dysfunction and related diseases is required.

### RIG-I in Cardiac Cell Pathology

Cell death including apoptosis, necrosis, and pyroptosis, are well-documented in heart disease (72). A previous study showed that RIG-I activator TNF-α upregulated the expression of RIP3, which was sufficient to induce necroptosis of cardiomyocytes during myocardial infarction (73). However, another study reported that TNF-α played a protective role in the early-stage of myocardial infarction in line with the regulation of autophagy and apoptosis (74). As for the role of another RIG-I activator IFN-γ in cardiomyocyte death is unclear. Considering the importance of cardiomyocyte death under various insults and the established role of IFN-γ in cell death (75), it would be worthwhile to further explore the role of RIG-I in IFN-γ associated-cardiomyocyte death. In cardiac fibroblasts, stimulation of RIG-I promoted the production of pro-inflammatory cytokines such as IL-6 and IL-8, contributing to heart injury and cardiomyopathy (76). This evidence suggests a pathogenic role for RIG-I in heart disease. However, in a pressure overload-induced cardiac hypertrophy and heart failure model, the RIG-I signaling pathway mediated the protective role of ADRB3 depletion by enhancing the innate immune response in the heart (77). Another study demonstrated the remodeling of scar fibroblasts into cardiomyocytes and thereby defined the protective role of RIG-I in heart repair. Hu et al. observed that a stabilized RNA, ICR2, increased the level of cardiomyocyte-specific genes in reprogrammed “fibroblasts” and enhanced their ability to differentiate into cardiomyocytes *via* the RIG-I and TLR3 pathways (78). In addition to the discrepancy in the above findings, the role of RIG-I in cell senescence is also controversial. Some studies have reported that RIG-I mediates senescence-associated inflammation (79, 80), while another study suggested that RIG-I inhibited cellular senescence by negatively regulating the integrin β3/p38 MAPK pathway (81). Therefore, the role of RIG-I in cardiac cell senescence requires further investigation. Overall, these controversial findings related to the role of RIG-I in cardiac cells under pathological conditions could be due to heterogeneity in the experimental settings and pathological conditions. Further studies are required to better define the function and underlying mechanisms of RIG-I in cardiac cell injuries.

### RIG-I in Macrophage Activation

Macrophage activation is not only involved in the innate immune system but also in immune-related cardiovascular diseases. RIG-I plays an important role in the antiviral innate immune response by inducing the production of type I IFN and pro-inflammatory cytokines in macrophages, as previously described. Imaizumi et al. found that RIG-I was expressed in macrophages of human atherosclerotic lesions, indicating that RIG-I may play a role in the differentiation and activation of macrophages in atherosclerosis (16). Another study found that RIG-I was significantly upregulated in LPS-stimulated primary human monocytes infected with dengue virus (DENV), resulting in vascular injury (82). These studies demonstrate that RIG-I expressed in macrophages participates in vascular injury and atherosclerosis. However, the function and related mechanism of RIG-I in macrophage activation-mediated cardiovascular diseases remain unclear and need to be explored.

### RIG-I in Cardiovascular Diseases

It is well known that inflammation plays a critical role in eliminating viruses and repairing damaged tissues. However, chronic inflammation often induces organ injury and triggers the onset of various diseases, including cardiovascular disease. Emerging studies have shown that RIG-I is involved in the pathogenesis of cardiovascular diseases.

### RIG-I in Atherosclerosis

Atherosclerosis is a progressive inflammatory disorder of the arterial wall that underlies hypertension, heart attack, and stroke (83). The study by Imaizumi et al. revealed RIG-I expression in foamy macrophages within atherosclerotic lesions, as well as IFN-gamma-induced RIG-I expression in macrophages, thereby suggesting the effect of RIG-I on the regulation of differentiation and activation of macrophages and induction of atherosclerosis (16). Another study showed that enhanced expression of RIG-I correlated with augmented lesions in atherosclerosis induced by organic pollutants (84).Wang’s study further revealed that 25-hydroxycholesterol induced higher expression of RIG-I in endothelial cells and macrophages, thereby contributing to atherosclerotic inflammation (20). RIG-I or MAVS deficiency reduced osteogenic signals in aortic vascular smooth muscle (VSM). Moreover, Blockage of RIG-I/MAVS signaling decreased aortic calcium accumulation in MAVS-deficient LDLR^-/-^ mice (70). These results provide new insights into the role of RIG-I in the pathogenesis of atherosclerosis and its therapeutic potential.

### RIG-I in Abdominal Aortic Aneurysm

Abdominal aortic aneurysm (AAA) is an inflammatory vascular disease that is common in the elderly. AAA is characterized by an inflammatory immune response and abdominal aorta dilation (85). The RIG-I gene expression in the aortic wall and blood of patients with AAA has been investigated. A previous study reported that RIG-I mRNA levels were enhanced in the circulation of patients with AAA compared with that in healthy subjects. RIG-I appears to be a promising biomarker for diagnosis and disease progression of AAA (86). Evidence from Ang II-induced AAA mouse models also revealed elevated RIG-I mRNA and protein levels. Moreover, animal experiments have shown that RIG-I overexpression by a lentivirus expression system resulted in the apoptosis of SMCs, which promoted AAA progression (71). The above studies suggest that RIG-I could serve as a promising biomarker for predicting the disease progression of AAA. Inhibition of this pathogenic signaling might be beneficial in retarding the progression of AAA.6

### RIG-I in Cardiac Dysfunction

Heart failure (HF) is one of the leading causes of death worldwide. Hypertensive heart disease, dilated cardiomyopathy, ischemic heart disease, and chronic obstructive pulmonary disease are the main causes of HF. Cardiac hypertrophy is the primary pathological change in hypertensive heart disease, however, its underlying molecular mechanisms remain unknown. Using an animal model with transverse aortic constriction (TAC), the authors observed that enhanced expression of RIG-I mediated the protective role of ADRB3 depletion in cardiac hypertrophy and heart failure (77). Such protection can be mediated by the enhancement of the innate immune response. In dilated cardiomyopathy, *in vitro* cell experiments showed that activation of RIG-I leads to higher production of pro-inflammatory cytokines such as IL-6 and IL-8, in human cardiac fibroblasts (76). This suggests the inflammatory function of RIG-I in the progression of dilated cardiomyopathy. Another study showed that ICR2 (a stabilized RNA) enhanced the ability of cardiac fibroblasts to reprogram into cardiomyocytes *via* the RIG-I pathway without inducing inflammatory events (78). These results reveal the controversial role of RIG-I in cardiac dysfunction caused by different stimuli and requires further research to validate the phenotypes and underlying mechanisms.

### RIG-I in Other Cardiovascular Diseases

Coronary artery disease (CAD) is a heart disease with a high morbidity rate. A study aimed at identifying potential biomarkers of CAD progression showed that genes enriched in the RIG-I-like receptor signaling pathway were possible candidates (87), and may be involved in the pathology of CAD. Although direct evidence of the role of RIG-I in hypertension is absent, RIG-I-like receptors might be involved in the pathological process of Ang II-induced hypertension (88). Considering the importance of inflammation in cardiovascular injury and the established role of RIG-I in inflammation, it would be worthwhile to further explore the role of RIG-I in CAD, hypertension, and other cardiovascular diseases.

**Figure 2:**
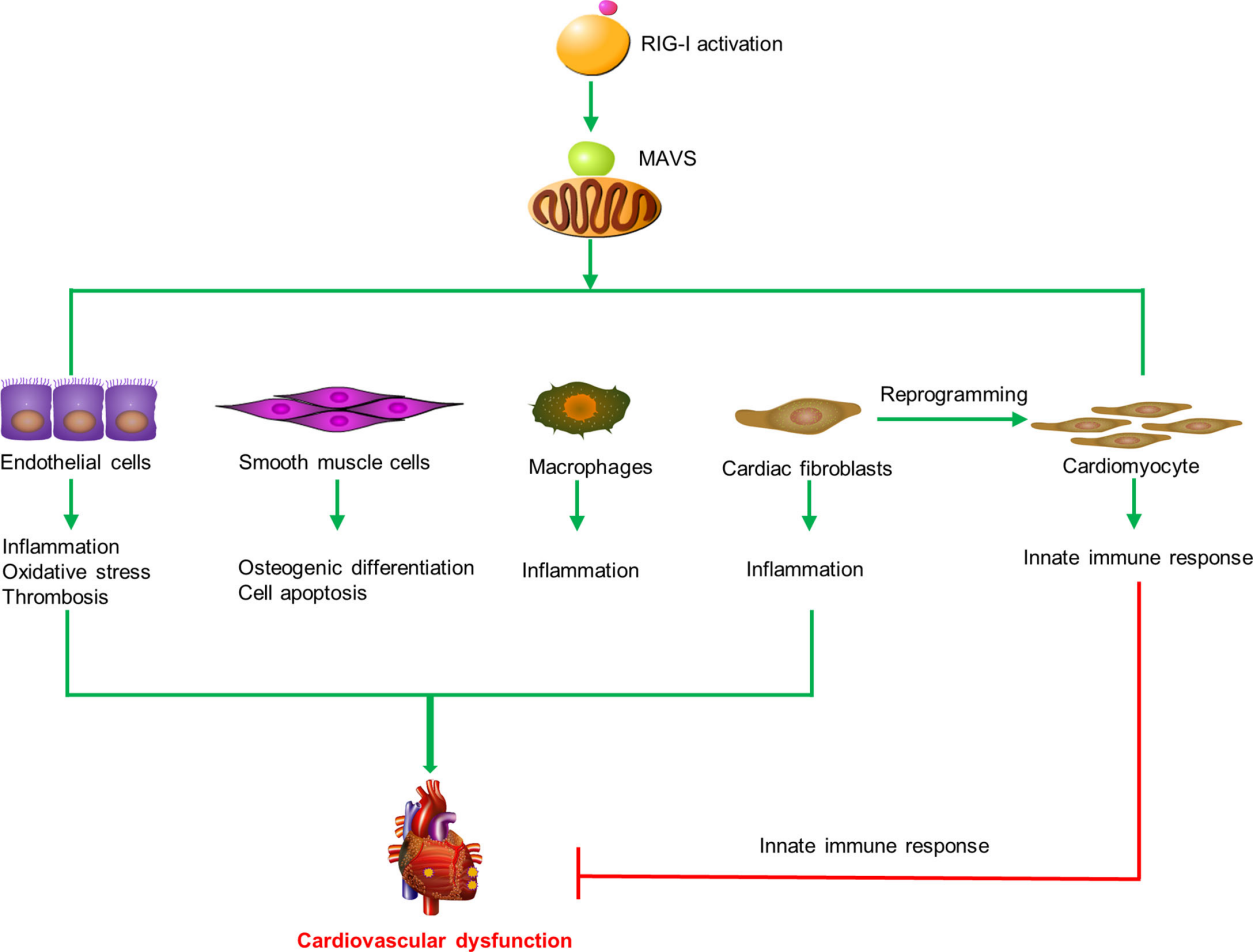
RIG-I triggers the innate immune response and participates in cardiovascular diseases. RIG-I is activated by activators and interacts with MAVS to trigger inflammatory responses, cell apoptosis, oxidative stress, prothrombotic proteins expression, osteogenic differentiation in macrophages, endothelial cells, smooth muscle cells, and cardiac fibroblasts to promote cardiovascular dysfunction. Otherwise, RIG-I activation mediates the remodeling of scar fibroblasts into cardiomyocytes and prevents cardiac hypertrophy by enhancing the innate immune response.

In summary, these results indicate that RIG-I plays diverse roles in cardiovascular diseases by inducing endothelial injury, SMC apoptosis, reprogramming of heart fibroblasts, and macrophage activation (**Figure 2**).

## Conclusion and Future Perspective

Accumulating evidence highlights the critical role of RIG-I in the innate immune and inflammatory responses involved in the pathogenesis of cardiovascular disease and this review adds substantial knowledge to existing literature. The role of RIG-I in the pathogenesis of cardiovascular disease, and the potential of RIG-I signaling as a biomarker for predicting the occurrence and progression of cardiovascular diseases has been established. However, more studies are required to validate the phenotypes of RIG-I in different cardiovascular diseases, as well as the underlying mechanisms. The development and application of RIG-I agonists and inhibitors could provide novel therapeutics that target the RIG-I signaling pathway for the treatment of cardiovascular diseases.

## Author Contributions

WX, HC and ZJ conducted the review, and WX, HW, and JY wrote the manuscript. XG and WS contributed to the revision of the manuscript. All the authors have reviewed and approved the manuscript.

## Funding

This study was supported by a grant from the National Natural Science Foundation of China (no. 82070760, 82070701, 81600352,82100779), Jiangsu Provincial Maternal and Child Health Research Institute (no. JSFY202105); grants from the Nanjing National Commission on Health and Family Planning (no. ZKX19042 and YKK18146).

## Conflict of Interest

The authors declare that the research was conducted in the absence of any commercial or financial relationships that could be construed as potential conflicts of interest.

## Publisher’s Note

All claims expressed in this article are solely those of the authors and do not necessarily represent those of their affiliated organizations, or those of the publisher, the editors and the reviewers. Any product that may be evaluated in this article, or claim that may be made by its manufacturer, is not guaranteed or endorsed by the publisher.

## References

[1] Matsukawa A Hogaboam CM Lukacs NW Kunkel SL. Chemokines and Innate Immunity. Rev Immunogenet (2000) 2(3):339–58. 11256744

[2] Lobo-Silva D Carriche GM Castro AG Roque S Saraiva M. Balancing the Immune Response in the Brain: IL-10 and its Regulation. J Neuroinflamm (2016) 13(1):297. doi: 10.1186/s12974-016-0763-8 PMC5121946 27881137

[3] McMaster WG Kirabo A Madhur MS Harrison DG. Inflammation, Immunity, and Hypertensive End-Organ Damage. Circ Res (2015) 116(6):1022–33. doi: 10.1161/CIRCRESAHA.116.303697 PMC4535695 25767287

[4] Ringseis R Eder K Mooren FC Krüger K. Metabolic Signals and Innate Immune Activation in Obesity and Exercise. Exerc Immunol Rev (2015) 21:58–68. 25825956

[5] Kawai T Akira S. The Role of Pattern-Recognition Receptors in Innate Immunity: Update on Toll-Like Receptors. Nat Immunol (2010) 11(5):373–84. doi: 10.1038/ni.1863 20404851

[6] Walsh D McCarthy J O'Driscoll C Melgar S. Pattern Recognition Receptors–Molecular Orchestrators of Inflammation in Inflammatory Bowel Disease. Cytokine Growth Factor Rev (2013) 24(2):91–104. doi: 10.1016/j.cytogfr.2012.09.003 23102645

[7] Rehwinkel J Reis e Sousa C. RIGorous Detection: Exposing Virus Through RNA Sensing. Science (2010) 327(5963):284–6. doi: 10.1126/science.1185068 20075242

[8] Errett JS Gale M. Emerging Complexity and New Roles for the RIG-I-Like Receptors in Innate Antiviral Immunity. Virol Sin (2015) 30(3):163–73. doi: 10.1007/s12250-015-3604-5 PMC7090589 25997992

[9] McNamara S Nichol JN Wang H Miller WH Jr. Targeting PKC Delta-Mediated Topoisomerase II Beta Overexpression Subverts the Differentiation Block in a Retinoic Acid-Resistant APL Cell Line. Leukemia (2010) 24(4):729–39. doi: 10.1038/leu.2010.27 20200558

[10] Kell AM Gale M Jr. RIG-I in RNA Virus Recognition. Virology (2015) 479-(480):110–21. doi: 10.1016/j.virol.2015.02.017 PMC4424084 25749629

[11] Weber M Weber F. RIG-I-Like Receptors and Negative-Strand RNA Viruses: RLRly Bird Catches Some Worms. Cytokine Growth Factor Rev (2014) 25(5):621–8. doi: 10.1016/j.cytogfr.2014.05.004 PMC7108359 24894317

[12] Carrión M Juarranz Y Pérez-García S Jimeno R Pablos JL Gomariz RP. RNA Sensors in Human Osteoarthritis and Rheumatoid Arthritis Synovial Fibroblasts: Immune Regulation by Vasoactive Intestinal Peptide. Arthritis Rheum (2011) 63(6):1626–36. doi: 10.1002/art.30294 21337319

[13] Funke B Lasitschka F Roth W Penzel R Meuer S Saile M. Selective Downregulation of Retinoic Acid-Inducible Gene I Within the Intestinal Epithelial Compartment in Crohn's Disease. Inflammation Bowel Dis (2011) 17(9):1943–54. doi: 10.1002/ibd.21572 21830273

[14] Hou J Zhou Y Zheng Y Fan J Zhou W Ng IO. Hepatic RIG-I Predicts Survival and Interferon-Alpha Therapeutic Response in Hepatocellular Carcinoma. Cancer Cell (2014) 25(1):49–63. doi: 10.1016/j.ccr.2013.11.011 24360797

[15] Imaizumi T Tanaka H Tajima A Tsuruga K Oki E Sashinami H. Retinoic Acid-Inducible Gene-I (RIG-I) is Induced by IFN-{Gamma} in Human Mesangial Cells in Culture: Possible Involvement of RIG-I in the Inflammation in Lupus Nephritis. Lupus (2010) 19(7):830–6. doi: 10.1177/0961203309360540 20167631

[16] Imaizumi T Yagihashi N Kubota K Yoshida H Sakaki H Yagihashi S. Expression of Retinoic Acid-Inducible Gene-I (RIG-I) in Macrophages: Possible Involvement of RIG-I in Atherosclerosis. J Atheroscler Thromb (2007) 14(2):51–5. doi: 10.5551/jat.14.51 17485888

[17] Jiang LJ Zhang NN Ding F Li XY Chen L Zhang HX. RA-Inducible Gene-I Induction Augments STAT1 Activation to Inhibit Leukemia Cell Proliferation. Proc Natl Acad Sci USA (2011) 108(5):1897–902. doi: 10.1073/pnas.1019059108 PMC3033283 21224412

[18] Tsuruga K Aizawa T Watanabe S Tsugawa K Yoshida H Imaizumi T. Expressions of mRNA for Innate Immunity-Associated Functional Molecules in Urinary Sediment in Immunoglobulin A Nephropathy. Nephrol (Carlton) (2015) 20(12):916–21. doi: 10.1111/nep.12533 26058859

[19] Tan YQ Li J Chen HW. Epac, a Positive or Negative Signaling Molecule in Cardiovascular Diseases. BioMed Pharmacother (2022) 148:112726. doi: 10.1016/j.biopha.2022.112726 35183995

[20] Wang F Xia W Liu F Li J Wang G Gu J. Interferon Regulator Factor 1/Retinoic Inducible Gene I (IRF1/RIG-I) Axis Mediates 25-Hydroxycholesterol-Induced Interleukin-8 Production in Atherosclerosis. Cardiovasc Res (2012) 93(1):190–9. doi: 10.1093/cvr/cvr260 21979142

[21] Jang MA Kim EK Now H Nguyen NT Kim WJ Yoo JY. Mutations in DDX58, Which Encodes RIG-I, Cause Atypical Singleton-Merten Syndrome. Am J Hum Genet (2015) 96(2):266–74. doi: 10.1016/j.ajhg.2014.11.019 PMC4320253 25620203

[22] Batool M Kim MS Choi S. Structural Insights Into the Distinctive RNA Recognition and Therapeutic Potentials of RIG-I-Like Receptors. Med Res Rev (2022) 42(1):399–425. doi: 10.1002/med.21845 34287999

[23] Ferrage F Dutta K Nistal-Villán E Patel JR Sánchez-Aparicio MT De Ioannes P. Structure and Dynamics of the Second CARD of Human RIG-I Provide Mechanistic Insights Into Regulation of RIG-I Activation. Structure (2012) 20(12):2048–61. doi: 10.1016/j.str.2012.09.003 PMC3625992 23063562

[24] Brisse M Ly H. Comparative Structure and Function Analysis of the RIG-I-Like Receptors: RIG-I and MDA5. Front Immunol (2019) 10:1586. doi: 10.3389/fimmu.2019.01586 31379819 PMC6652118

[25] Myong S Cui S Cornish PV Kirchhofer A Gack MU Jung JU. Cytosolic Viral Sensor RIG-I is a 5'-Triphosphate-Dependent Translocase on Double-Stranded RNA. Science (2009) 323(5917):1070–4. doi: 10.1126/science.1168352 PMC3567915 19119185

[26] Thoresen D Wang W Galls D Guo R Xu L Pyle AM. The Molecular Mechanism of RIG-I Activation and Signaling. Immunol Rev (2021) 304(1):154–68. doi: 10.1111/imr.13022 PMC9293153 34514601

[27] Hornung V Ellegast J Kim S Brzózka K Jung A Kato H. 5'-Triphosphate RNA is the Ligand for RIG-I. Science (2006) 314(5801):994–7. doi: 10.1126/science.1132505 17038590

[28] Kato H Takeuchi O Sato S Yoneyama M Yamamoto M Matsui K. Differential Roles of MDA5 and RIG-I Helicases in the Recognition of RNA Viruses. Nature (2006) 441(7089):101–5. doi: 10.1038/nature04734 16625202

[29] Schlee M Roth A Hornung V Hagmann CA Wimmenauer V Barchet W. Recognition of 5' Triphosphate by RIG-I Helicase Requires Short Blunt Double-Stranded RNA as Contained in Panhandle of Negative-Strand Virus. Immunity (2009) 31(1):25–34. doi: 10.1016/j.immuni.2009.05.008 19576794 PMC2824854

[30] Anchisi S Guerra J Garcin D. RIG-I ATPase Activity and Discrimination of Self-RNA Versus non-Self-RNA. mBio (2015) 6(2):e02349. doi: 10.1128/mBio.02349-14 25736886 PMC4358010

[31] Devarkar SC Wang C Miller MT Ramanathan A Jiang F Khan AG. Structural Basis for M7g Recognition and 2'-O-Methyl Discrimination in Capped RNAs by the Innate Immune Receptor RIG-I. Proc Natl Acad Sci USA (2016) 113(3):596–601. doi: 10.1073/pnas.1515152113 26733676 PMC4725518

[32] Schuberth-Wagner C Ludwig J Bruder AK Herzner AM Zillinger T Goldeck M. A Conserved Histidine in the RNA Sensor RIG-I Controls Immune Tolerance to N1-2'o-Methylated Self RNA. Immunity (2015) 43(1):41–51. doi: 10.1016/j.immuni.2015.06.015 26187414 PMC7128463

[33] Chung H Calis JJA Wu X Sun T Yu Y Sarbanes SL. Human ADAR1 Prevents Endogenous RNA From Triggering Translational Shutdown. Cell (2018) 172(4):811–824.e14. doi: 10.1016/j.cell.2017.12.038 29395325 PMC5831367

[34] Lassig C Lammens K Gorenflos López JL Michalski S Fettscher O Hopfner KP. Unified Mechanisms for Self-RNA Recognition by RIG-I Singleton-Merten Syndrome Variants. Elife (2018) 7:e38958. doi: 10.7554/eLife.38958 30047865 PMC6086658

[35] Yoneyama M Kikuchi M Matsumoto K Imaizumi T Miyagishi M Taira K. Shared and Unique Functions of the DExD/H-Box Helicases RIG-I, MDA5, and LGP2 in Antiviral Innate Immunity. J Immunol (2005) 175(5):2851–8. doi: 10.4049/jimmunol.175.5.2851 16116171

[36] Pichlmair A Schulz O Tan CP Näslund TI Liljeström P Weber F. RIG-I-Mediated Antiviral Responses to Single-Stranded RNA Bearing 5'-Phosphates. Science (2006) 314(5801):997–1001. doi: 10.1126/science.1132998 17038589

[37] Saito T Owen DM Jiang F Marcotrigiano J Gale Jr M. Innate Immunity Induced by Composition-Dependent RIG-I Recognition of Hepatitis C Virus RNA. Nature (2008) 454(7203):523–7. doi: 10.1038/nature07106 PMC2856441 18548002

[38] Yoneyama M Kikuchi M Natsukawa T Shinobu N Imaizumi T Miyagishi M. The RNA Helicase RIG-I has an Essential Function in Double-Stranded RNA-Induced Innate Antiviral Responses. Nat Immunol (2004) 5(7):730–7. doi: 10.1038/ni1087 15208624

[39] Tigano M Vargas DC Tremblay-Belzile S Fu Y Sfeir A. Nuclear Sensing of Breaks in Mitochondrial DNA Enhances Immune Surveillance. Nature (2021) 591(7850):477–81. doi: 10.1038/s41586-021-03269-w 33627873

[40] Hemmi H Takeuchi O Kawai T Kaisho T Sato S Sanjo H. A Toll-Like Receptor Recognizes Bacterial DNA. Nature (2000) 408(6813):740–5. doi: 10.1038/35047123 11130078

[41] Hornung V Ablasser A Charrel-Dennis M Bauernfeind F Horvath G Caffrey DR. AIM2 Recognizes Cytosolic dsDNA and Forms a Caspase-1-Activating Inflammasome With ASC. Nature (2009) 458(7237):514–8. doi: 10.1038/nature07725 PMC2726264 19158675

[42] Sun L Wu J Du F Chen X Chen ZJ. Cyclic GMP-AMP Synthase is a Cytosolic DNA Sensor That Activates the Type I Interferon Pathway. Science (2013) 339(6121):786–91. doi: 10.1126/science.1232458 PMC3863629 23258413

[43] Choi MK Wang Z Ban T Yanai H Lu Y Koshiba R. A Selective Contribution of the RIG-I-Like Receptor Pathway to Type I Interferon Responses Activated by Cytosolic DNA. Proc Natl Acad Sci U S A (2009) 106(42):17870–5. doi: 10.1073/pnas.0909545106 PMC2764914 19805092

[44] Imaizumi T Aratani S Nakajima T Carlson M Matsumiya T Tanji K. Retinoic Acid-Inducible Gene-I is Induced in Endothelial Cells by LPS and Regulates Expression of COX-2. Biochem Biophys Res Commun (2002) 292(1):274–9. doi: 10.1006/bbrc.2002.6650 11890704

[45] Imaizumi T Hatakeyama M Yamashita K Yoshida H Ishikawa A Taima K. Interferon-Gamma Induces Retinoic Acid-Inducible Gene-I in Endothelial Cells. Endothelium (2004) 11(3-4):169–73. doi: 10.1080/10623320490512156 15370293

[46] Sakaki H Imaizumi T Matsumiya T Kusumi A Nakagawa H Kubota K. Retinoic Acid-Inducible Gene-I is Induced by Interleukin-1beta in Cultured Human Gingival Fibroblasts. Oral Microbiol Immunol (2005) 20(1):47–50. doi: 10.1111/j.1399-302X.2005.00181.x 15612946

[47] Imaizumi T Matsumiya T Yoshida H Naraoka T Uesato R Ishibashi Y. Tumor-Necrosis Factor-Alpha Induces Retinoic Acid-Inducible Gene-I in Rheumatoid Fibroblast-Like Synoviocytes. Immunol Lett (2009) 122(1):89–93. doi: 10.1016/j.imlet.2008.12.005 19126414

[48] Jordan AM Tatum R Ahmad D Patel SV Maynes EJ Weber MP. Infective Endocarditis Following Heart Transplantation: A Systematic Review. Transplant Rev (Orlando) (2022) 36(1):100672. doi: 10.1016/j.trre.2021.100672 34826752

[49] Verbsky J. Genetic Defects That Predispose to Serious Viral Infections. Crit Care Clin (2022) 38(2):443–53. doi: 10.1016/j.ccc.2021.11.012 35369956

[50] Zendehdel A Roham M. Role of Helicobacter Pylori Infection in the Manifestation of Old Age-Related Diseases. Mol Genet Genomic Med (2020) 8(4):e1157. doi: 10.1002/mgg3.1157 32067423 PMC7196471

[51] Merino I de la Fuente A Domínguez-Gil M Eiros JM Tedim AP Bermejo-Martín JF. Digital PCR Applications for the Diagnosis and Management of Infection in Critical Care Medicine. Crit Care (2022) 26(1):63. doi: 10.1186/s13054-022-03948-8 35313934 PMC8935253

[52] Seth RB Sun L Ea CK Chen ZJ. Identification and Characterization of MAVS, a Mitochondrial Antiviral Signaling Protein That Activates NF-kappaB and IRF 3. Cell (2005) 122(5):669–82. doi: 10.1016/j.cell.2005.08.012 16125763

[53] Kumar H Kawai T Kato H Sato S Takahashi K Coban C. Essential Role of IPS-1 in Innate Immune Responses Against RNA Viruses. J Exp Med (2006) 203(7):1795–803. doi: 10.1084/jem.20060792 PMC2118350 16785313

[54] Fitzgerald KA McWhirter SM Faia KL Rowe DC Latz E Golenbock DT. IKKepsilon and TBK1 are Essential Components of the IRF3 Signaling Pathway. Nat Immunol (2003) 4(5):491–6. doi: 10.1038/ni921 12692549

[55] Hayden MS Ghosh S. Signaling to NF-kappaB. Genes Dev (2004) 18(18):2195–224. doi: 10.1101/gad.1228704 15371334

[56] Ablasser A Bauernfeind F Hartmann G Latz E Fitzgerald KA Hornung V. RIG-I-Dependent Sensing of Poly(Da:Dt) Through the Induction of an RNA Polymerase III-Transcribed RNA Intermediate. Nat Immunol (2009) 10(10):1065–72. doi: 10.1038/ni.1779 PMC3878616 19609254

[57] Gimbrone M A Jr. Garcia-Cardena G. Endothelial Cell Dysfunction and the Pathobiology of Atherosclerosis. Circ Res (2016) 118(4):620–36. doi: 10.1161/CIRCRESAHA.115.306301 PMC4762052 26892962

[58] Zimmerman GA McIntyre TM Prescott SM. Adhesion and Signaling in Vascular Cell–Cell Interactions. J Clin Invest (1996) 98(8):1699–702. doi: 10.1172/JCI118967 PMC507606 8878418

[59] Catalina MD Estess P Siegelman MH. Selective Requirements for Leukocyte Adhesion Molecules in Models of Acute and Chronic Cutaneous Inflammation: Participation of E- and P- But Not L-Selectin. Blood (1999) 93(2):580–9. doi: 10.1182/blood.V93.2.580 9885219

[60] Asada Y Yamashita A Sato Y Hatakeyama K. Thrombus Formation and Propagation in the Onset of Cardiovascular Events. J Atheroscler Thromb (2018) 25(8):653–64. doi: 10.5551/jat.RV17022 PMC6099067 29887539

[61] da Conceição TM Rust NM Berbel AC Martins NB do NascimentoSantos CA Da Poian A. Essential Role of RIG-I in the Activation of Endothelial Cells by Dengue Virus. Virology (2013) 435(2):281–92. doi: 10.1016/j.virol.2012.09.038 23089253

[62] Shi F Li Q Liu S Liu F Wang J Cui D. Porcine Circovirus Type 2 Upregulates Endothelial-Derived IL-8 Production in Porcine Iliac Artery Endothelial Cells via the RIG-I/MDA-5/MAVS/JNK Signaling Pathway. BMC Vet Res (2020) 16(1):265. doi: 10.1186/s12917-020-02486-1 32727484 PMC7392700

[63] Asdonk T Motz I Werner N Coch C Barchet W Hartmann G. Endothelial RIG-I Activation Impairs Endothelial Function. Biochem Biophys Res Commun (2012) 420(1):66–71. doi: 10.1016/j.bbrc.2012.02.116 22402283

[64] Ando M Matsumoto T Taguchi K Kobayashi T. Poly (I:C) Impairs NO Donor-Induced Relaxation by Overexposure to NO via the NF-Kappa B/iNOS Pathway in Rat Superior Mesenteric Arteries. Free Radic Biol Med (2017) 112:553–66. doi: 10.1016/j.freeradbiomed.2017.08.027 28870522

[65] Dayang EZ Plantinga J Ter Ellen B van Meurs M Molema G Moser J. Identification of LPS-Activated Endothelial Subpopulations With Distinct Inflammatory Phenotypes and Regulatory Signaling Mechanisms. Front Immunol (2019) 10:1169. doi: 10.3389/fimmu.2019.01169 31178871 PMC6543489

[66] Imaizumi T Yamashita K Taima K Ishikawa A Yoshida H Satoh K. Effect of Peroxisome Proliferator-Activated Receptor-Gamma Ligands on the Expression of Retinoic Acid-Inducible Gene-I in Endothelial Cells Stimulated With Lipopolysaccharide. Prostaglandins Other Lipid Mediat (2005) 78(1-4):46–54. doi: 10.1016/j.prostaglandins.2005.02.006 16303604

[67] Gaitzsch E Czermak T Ribeiro A Heun Y Bohmer M Merkle M. Double-Stranded DNA Induces a Prothrombotic Phenotype in the Vascular Endothelium. Sci Rep (2017) 7(1):1112. doi: 10.1038/s41598-017-01148-x 28442771 PMC5430798

[68] Ross R. Atherosclerosis–an Inflammatory Disease. N Engl J Med (1999) 340(2):115–26. doi: 10.1056/NEJM199901143400207 9887164

[69] Imaizumi T Yagihashi N Hatakeyama M Yamashita K Ishikawa A Taima K. Expression of Retinoic Acid-Inducible Gene-I in Vascular Smooth Muscle Cells Stimulated With Interferon-Gamma. Life Sci (2004) 75(10):1171–80. doi: 10.1016/j.lfs.2004.01.030 15219805

[70] Ramachandran B Stabley JN Cheng SL Behrmann AS Gay A Li L. A GTPase-Activating Protein-Binding Protein (G3BP1)/antiviral Protein Relay Conveys Arteriosclerotic Wnt Signals in Aortic Smooth Muscle Cells. J Biol Chem (2018) 293(21):7942–68. doi: 10.1074/jbc.RA118.002046 PMC5971440 29626090

[71] Le T He X Huang J Liu S Bai Y Wu K. Knockdown of Long Noncoding RNA GAS5 Reduces Vascular Smooth Muscle Cell Apoptosis by Inactivating EZH2-Mediated RIG-I Signaling Pathway in Abdominal Aortic Aneurysm. J Transl Med (2021) 19(1):466. doi: 10.1186/s12967-021-03023-w 34781960 PMC8594130

[72] Del Re DP Amgalan D Linkermann A Liu Q Kitsis RN. Fundamental Mechanisms of Regulated Cell Death and Implications for Heart Disease. Physiol Rev (2019) 99(4):1765–817. doi: 10.1152/physrev.00022.2018 PMC6890986 31364924

[73] Luedde M Lutz M Carter N Sosna J Jacoby C Vucur M. RIP3, a Kinase Promoting Necroptotic Cell Death, Mediates Adverse Remodelling After Myocardial Infarction. Cardiovasc Res (2014) 103(2):206–16. doi: 10.1093/cvr/cvu146 24920296

[74] Wang X Guo Z Ding Z Mehta JL. Inflammation, Autophagy, and Apoptosis After Myocardial Infarction. J Am Heart Assoc (2018) 7(9):e008024. doi: 10.1161/JAHA.117.008024 29680826 PMC6015297

[75] Araujo A Safronova A Burger E López-Yglesias A Giri S Camanzo ET. IFN-Gamma Mediates Paneth Cell Death via Suppression of mTOR. Elife (2021) 10:e60478. doi: 10.7554/eLife.60478. 34633285 PMC8570691

[76] Li Z Nguyen TT Valaperti A. Human Cardiac Fibroblasts Produce Pro-Inflammatory Cytokines Upon TLRs and RLRs Stimulation. Mol Cell Biochem (2021) 476(9):3241–52. doi: 10.1007/s11010-021-04157-7 PMC8059428 33881711

[77] Wei X Zhang A Yang W Fang Y. Depletion of Beta3-Adrenergic Receptor Relieves Pressure Overload-Induced Cardiac Hypertrophy and Heart Failure via Enhancing Innate Immune Response. BioMed Pharmacother (2021) 143:112194. doi: 10.1016/j.biopha.2021.112194 34563949

[78] Hu J Hodgkinson CP Pratt RE Lee J Sullenger BA Dzau VJ. Enhancing Cardiac Reprogramming via Synthetic RNA Oligonucleotides. Mol Ther Nucleic Acids (2021) 23:55–62. doi: 10.1016/j.omtn.2020.10.034 33335792 PMC7723775

[79] Liu F Wu S Ren H Gu J. Klotho Suppresses RIG-I-Mediated Senescence-Associated Inflammation. Nat Cell Biol (2011) 13(3):254–62. doi: 10.1038/ncb2167 21336305

[80] Zeng Y Wang PH Zhang M Du JR. Aging-Related Renal Injury and Inflammation are Associated With Downregulation of Klotho and Induction of RIG-I/NF-kappaB Signaling Pathway in Senescence-Accelerated Mice. Aging Clin Exp Res (2016) 28(1):69–76. doi: 10.1007/s40520-015-0371-y 25986237

[81] Zhao J Jiang X Yan L Lin J Guo H Yu S. Retinoic Acid Inducible Gene-I Slows Down Cellular Senescence Through Negatively Regulating the Integrin Beta3/P38 MAPK Pathway. Cell Cycle (2019) 18(23):3378–92. doi: 10.1080/15384101.2019.1677074 PMC6927694 31595820

[82] Kamaladasa A Gomes L Jeewandara C Shyamali NL Ogg GS Malavige GN. Lipopolysaccharide Acts Synergistically With the Dengue Virus to Induce Monocyte Production of Platelet Activating Factor and Other Inflammatory Mediators. Antiviral Res (2016) 133:183–90. doi: 10.1016/j.antiviral.2016.07.016 27476044

[83] Fenyo IM Gafencu AV. The Involvement of the Monocytes/Macrophages in Chronic Inflammation Associated With Atherosclerosis. Immunobiology (2013) 218(11):1376–84. doi: 10.1016/j.imbio.2013.06.005 23886694

[84] Shan Q Wang J Huang F Lv X Ma M Du Y. Augmented Atherogenesis in ApoE-Null Mice Co-Exposed to Polychlorinated Biphenyls and 2,3,7,8-Tetrachlorodibenzo-P-Dioxin. Toxicol Appl Pharmacol (2014) 276(2):136–46. doi: 10.1016/j.taap.2014.02.007 24582691

[85] Gao H Wang L Ren J Liu Y Liang S Zhang B. Interleukin 2 Receptor Subunit Beta as a Novel Hub Gene Plays a Potential Role in the Immune Microenvironment of Abdominal Aortic Aneurysms. Gene (2022) 827:146472. doi: 10.1016/j.gene.2022.146472 35381314

[86] Jablonska A Neumayer C Bolliger M Gollackner B Klinger M Paradowska E. Analysis of Host Toll-Like Receptor 3 and RIG-I-Like Receptor Gene Expression in Patients With Abdominal Aortic Aneurysm. J Vasc Surg (2018) 68(6S):39S–46S. doi: 10.1016/j.jvs.2017.10.087 29567028

[87] Wang L Hu J Zhou J Guo F Yao T Zhang L. Weighed Gene Coexpression Network Analysis Screens the Potential Long Noncoding RNAs and Genes Associated With Progression of Coronary Artery Disease. Comput Math Methods Med (2020) 2020:8183420. doi: 10.1155/2020/8183420 32695216 PMC7361886

[88] Lin QY Bai J Liu JQ Li HH. Angiotensin II Stimulates the Proliferation and Migration of Lymphatic Endothelial Cells Through Angiotensin Type 1 Receptors. Front Physiol (2020) 11:560170. doi: 10.3389/fphys.2020.560170 33013481 PMC7506107

